# Comparative analysis of the carrot miRNAome in response to salt stress

**DOI:** 10.1038/s41598-023-48900-0

**Published:** 2023-12-06

**Authors:** Kamil Szymonik, Magdalena Klimek-Chodacka, Aneta Lukasiewicz, Alicja Macko-Podgórni, Dariusz Grzebelus, Rafal Baranski

**Affiliations:** Department of Plant Biology and Biotechnology, Faculty of Biotechnology and Horticulture, University of Agriculture in Krakow, AL. Mickiewicza 21, 31-120 Kraków, Poland

**Keywords:** Plant sciences, Plant stress responses, Salt

## Abstract

Soil salinity adversely affects the yield and quality of crops, including carrot. During salt stress, plant growth and development are impaired by restricted water uptake and ion cytotoxicity, leading to nutrient imbalance and oxidative burst. However, the molecular mechanisms of the carrot plant response to salt stress remain unclear. The occurrence and expression of miRNAs that are potentially involved in the regulation of carrot tolerance to salinity stress were investigated. The results of small RNA sequencing revealed that salt-sensitive (DH1) and salt-tolerant (DLBA) carrot varieties had different miRNA expression profiles. A total of 95 miRNAs were identified, including 71 novel miRNAs, of which 30 and 23 were unique to DH1 and DLBA, respectively. The comparison of NGS and qPCR results allowed identification of two conserved and five novel miRNA involved in carrot response to salt stress, and which differentiated the salt-tolerant and salt-sensitive varieties. Degradome analysis supported by in silico-based predictions and followed by expression analysis of exemplary target genes pointed at genes related to proline, glutathione, and glutamate metabolism pathways as potential miRNA targets involved in salt tolerance, and indicated that the regulation of osmoprotection and antioxidant protection, earlier identified as being more efficient in the tolerant variety, may be controlled by miRNAs. Furthermore, potential miRNA target genes involved in chloroplast protection, signal transduction and the synthesis and modification of cell wall components were indicated in plants growing in saline soil.

## Introduction

Soil salinity is a significant problem for contemporary agriculture and the global food security. Approximately 20% of cultivated soil is salt-affected^1^ and more than 50% of all arable lands are potentially vulnerable to soil salinization caused by irrigation and climate changes by 2050^2^. Understanding the mechanisms of plant tolerance to soil salinity is necessary to support breeding of salt-tolerant varieties which remains a serious challenge especially in salt-sensitive crops such as carrot. Carrot is an important root vegetable cultivated on 1.13 million hectares worldwide, which production is close to 41 million tons^3^ and is one of the most important sources of pro-vitamin A (α-carotene and β-carotene) in the human diet^4^. Soil salinity highly contributes to the loss of carrot root yield and its market value^5^. Maas et al., 1986 reported that root yield declined 14% for every unit increase in salinity above the threshold of 1.0 dS/m, thus classified carrot as salt-sensitive^6^. The use of poor quality water for irrigation in conventional farming strengthens salt accumulation in the soil and, in consequence, reduces carrot yield^7,8^. Previous investigations showed that soil salinity affected carrot seed germination^5^, physiological processes^9^, and mineral balance^10^. Several genes involved in the plant response to salinity have been identified and two of them were introduced via genetic transformation to carrot. Genetically modified carrot callus and plants expressing the *Vigna aconitifolia* Δ^1^-pyrroline-5-carboxylate synthase (*P5CS*) gene accumulated more proline and showed higher level of salt tolerance than the non-transgenic control^11^. Transgenic carrot plants overexpressing *betaine aldehyde dehydrogenase* (*BADH*) grew well when watered with up to 400 mM NaCl solution while the control non-transgenic plants exhibited comparable growth and vitality in the presence of 100 mM NaCl^12^. Despite carrot is a glycophyte, some carrot varieties are cultivated in saline soil therefore they must have active mechanisms enhancing their tolerance^13^. Protoplasts isolated from such locally grown salt-tolerant Iranian variety were able to withstand elevated NaCl concentrations in the medium and developed into plants^14^. A salt-tolerant local variety (DLBA) of eastern type, grown in the saline soil performed much better than DH1, a doubled haploid line representing Nantes orange carrot of western type commonly cultivated in Europe and the USA. Although, all salt-stressed DLBA and DH1 plants had restricted growth, the DLBA plants had higher vigour, their biomass was halved while the biomass of DH1 plants was reduced to a third^10^. It was shown that DLBA plants had more efficient osmoprotective and antioxidative mechanisms in roots and leaves than DH1, in particular those related to proline content and glutathione activity^9^. A significant increase in the expression of Na^+^/H^+^ exchange antiporter *DcNHX4* gene was also found in the leaves of salt-stressed DLBA plants, in contrast to the lack of *DcNHX4* expression in DH1^10^. Such differential reaction of salt-tolerant and salt-sensitive carrot varieties was in agreement with the evidence that overexpression of *NHX* conferred salt tolerance in a wide range of plant species due to NHX role in maintaining cation homeostasis in the cell^15^. These reports indicated that salt tolerance found in some carrot landraces was genetically determined.

The expression of most genes may be mediated by microRNAs (miRNAs). Plant miRNAs are 20–24 nt-long, endogenous, non-coding RNAs (ncRNAs) participating in post-transcriptional regulatory mechanisms of gene expression by degrading target mRNAs^16^ or repressing their translation^17^. The miRNAs play important role in plant developmental and physiological processes. They participate in plant response to biotic^18^ and abiotic stresses such as drought, cold, heat, heavy metal toxicity, mineral nutrient deficiency^19^. Several reports indicated that miRNAs were involved in plant tolerance to salinity^20^. Lotfti et al. proposed a list of up- and down-regulated miRNAs involved in salt-stress response, classified to 35 miRNA families from 19 plant species^21^. Three physiological processes, i.e. antioxidant production, ABA response and auxin signalling were identified in the miRNA-dependent gene regulatory network in reaction to salinity. There has been little research to date regarding carrot miRNAs. An initial report indicated 17 carrot miRNAs belonging to 12 families^22^. Recently, 130 and 47 up- and down-regulated carrot miRNAs (dca-miRNAs), respectively, were identified as being involved in carotenoid and anthocyanin biosynthesis pathways in two carrot varieties^23^. To the best of our knowledge, there have been no reports on dca-miRNAs engagement in stress responses.

The aim of this work was to identify dca-miRNAs involved in carrot response to salt stress and to identify their potential targets in the carrot genome. For this purpose, we performed sRNA and degradome sequencing using RNA extracted from roots and leaves of salt-tolerant and salt-sensitive plants exposed to salt stress.

## Results

### Identification of miRNAs

To identify miRNAs involved in carrot response to salt stress, the Illumina technology was applied to sequence 24 small RNA libraries from leaves and roots of plants representing two carrot varieties, salt-tolerant DLBA and salt-sensitive DH1, grown in a highly saline and in the control soil. Approximately 10.4 billion raw reads of 10–44 nt sRNA were obtained from sequencing of all libraries and the reads were mapped to the carrot ncRNA database to filter out ncRNAs other than miRNAs. Up to 1.1% of ncRNAs (rRNA, tRNA, snRNA, snoRNA) were detected, with the rRNA category being the most abundant (97% of filtered out ncRNAs) (Supplementary file 1). After FastQC processing, clean reads of 18–30 nt were identified. The size distribution of redundant reads was similar in both carrot varieties except of the 22 nt length fraction, with approx. 23% higher abundance in DLBA regardless of the treatment; and the 24 nt length fraction in the leaves of salt-stressed DH1, where about 28% more reads were observed than in the leaves of salt-stressed DLBA (Supplementary file 2). The distribution of the total size of the unique reads were similar in all libraries. The most abundant were 24 nt-long followed by 21 nt–long small RNAs. In total, 95 miRNA candidates, represented by at least 300 reads in each replication, of at least one research group, were identified. Among them, 71 miRNAs were novel while 24 have been deposited in miRBase or sRNAanno databases. Of the latter, 11 miRNAs were attributed to carrot while the remaining 13 miRNAs were identified in other species (Supplementary file 3). Carrot miRNA precursors were predicted using MTide. Their lengths varied widely from 44 to 244 nt and over 51% of them ranged from 80 to 140 nt. In 84% of the precursors, the GC content was above 30% and reached the maximum of 54%. MIR genes were distributed on all carrot chromosomes with the highest number (18 MIR genes) assigned to chromosome 3 and the lowest (4 genes) to chromosome 8 (Supplementary file 3).

### Occurrence of miRNAs

**Figure 1:**
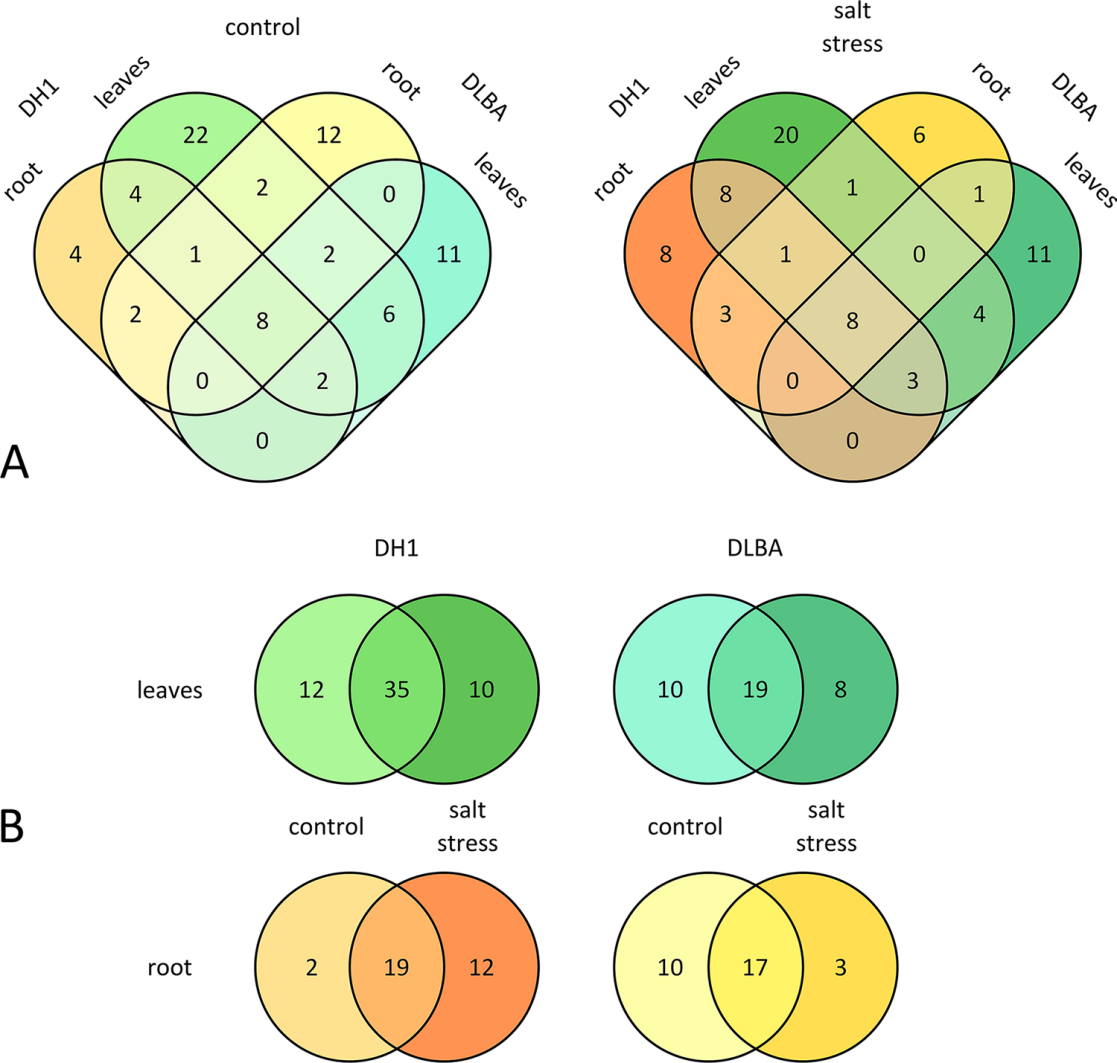
Distribution of dca-miRNAs candidates in accordance with the treatment (control/salt stress) (**A**), tissue (root/leaves) and carrot variety (DH1/DLBA) (**B**).

**Figure 2:**
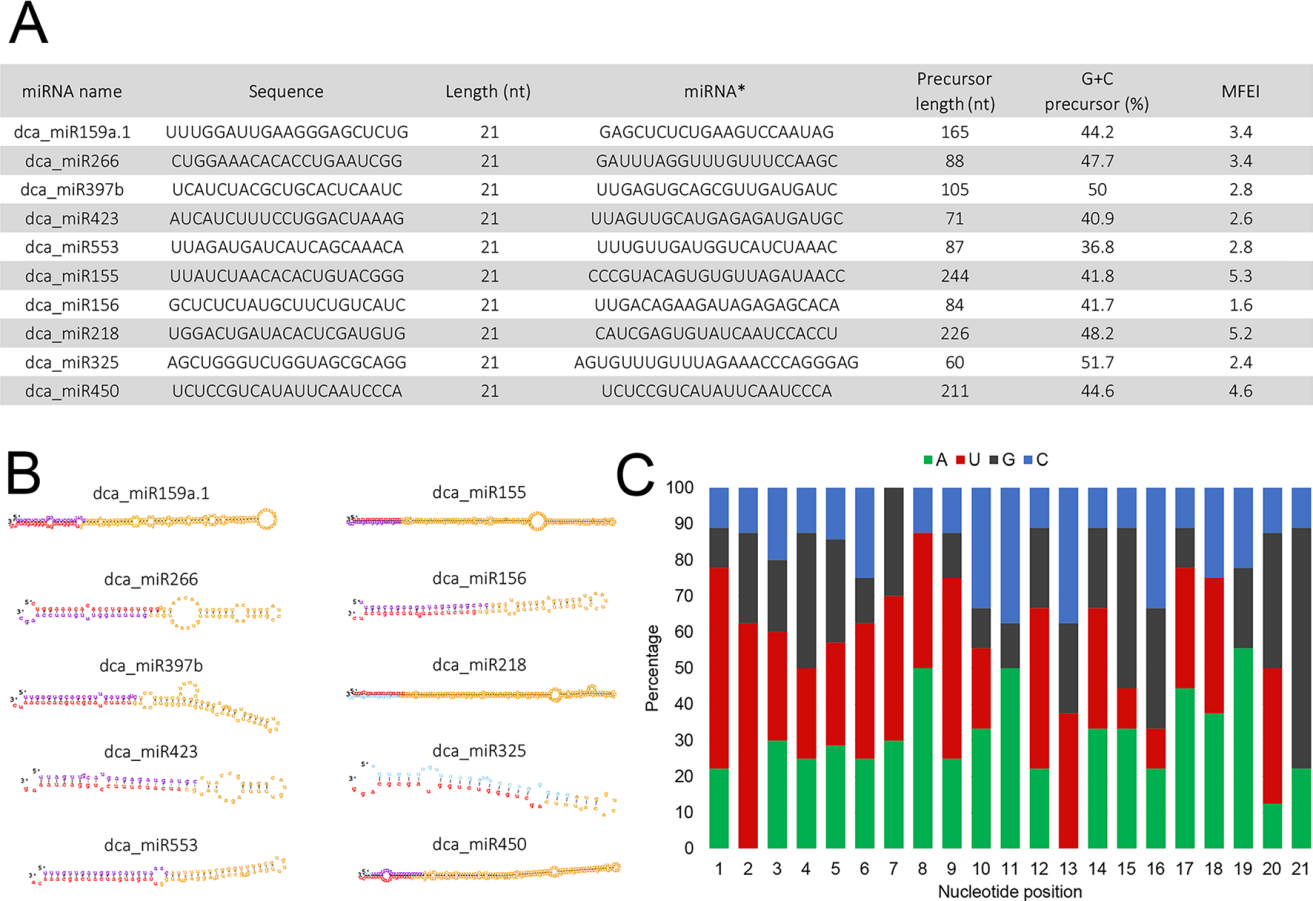
Carrot miRNA candidates chosen for expression validation by qPCR (**A**). Hairpin structures of carrot miRNAs precursors, as predicted by ViennaRNA (red—miRNA sequence, purple—miRNA*) (**B**). Relative nucleotide bias of miRNAs listed in 2A (**C**).

The comparison of DH1 and DLBA grown in the control conditions revealed that slightly more miRNAs were identified for DH1 (53 miRNAs) than for DLBA (46 miRNAs) (Fig. 1A). Only 23 miRNAs were common to both varieties, 30 miRNAs were unique to DH1 and 23 to DLBA. For DH1, 47 miRNAs were found in the leaves and 21 in the storage root; 32 miRNAs were found in the leaves only and 6 in the root only while 15 miRNAs were common to both plant parts. For DLBA, the number of miRNAs in the leaves was almost twice lower (29 miRNA) while in the root it was similar (27 miRNA) in comparison to DH1; of those 19 miRNAs were found in the leaves only and 17 in the root only while 10 miRNAs were common to both parts of DLBA plants. Regardless of the variety, twice more miRNAs were uniquely expressed in the leaves (39 miRNAs) than in the root (18 miRNAs) and 19 miRNAs were common to both plant parts. The salt treatment silenced the expression of 21 miRNA in the leaves or in the root in at least one variety and this effect was more evident for DLBA, in particular in the root. For DH1, 12 miRNAs in the leaves and two miRNAs in the root were silenced while for DLBA these numbers were ten and ten, respectively (Fig. 1B). The salt treatment also induced the expression of miRNAs not present in the control plants and this effect was more evident for DH1. Ten miRNAs were induced in the leaves and 12 in the root of DH1, while for DLBA, eight and three, respectively (Fig. 2B). Only eight miRNAs were identified in both varieties in both plant parts. Differential expression of miRNAs between the salt-stressed and control plants was also identified (Supplementary file 4). Fourteen and two of miRNAs were differentially expressed in the roots of DH1 and DLBA, respectively. Conversely, in the leaves there were less differentially expressed miRNAs in DH1 (8), and in DLBA (6). On average, twice more miRNAs were up-regulated than down-regulated in DH1, independent on the plant part. In contrast, in DLBA this ratio was reversed. The expression change fold values were also higher for DH1 than for DLBA. For DH1, the expression was up to sixfold lower and up to threefold higher in the salt-stressed plants than in control while for DLBA these change fold values were 2.0 and 2.5, respectively.

### Validation of identified miRNAs

Three conserved and seven novel miRNAs (Fig. 2A) were selected to validate the results of RNASeq data by qPCR. For that purpose, RNA samples were obtained in an independent experiment from salt-stressed plants grown in the following season. These miRNAs were 21 nt in length and their precursors had predicted hairpin structures (Fig. 2B) with minimum folding free energy index (MFEI) ranging from 1.6 to 5.3. They were characterized by a high total GC content (36–51%), AU nucleotides dominated at the 5' end and GC nucleotides at the 3' end (Fig. 2C). The most frequently occurring nucleotide at the first position was U representing 50% of all nucleotides at that position. At the expected cleavage site, located between 10 and 11 nt of miRNA, the most abundant nucleotides were A and C.

In qPCR reactions, fluorescent signals were detected for all ten miRNAs that experimentally validated expression of miRNAs identified based on bioinformatic analysis of the NGS data. Various expression levels were detected depending on variety, plant organ, and treatment (Supplementary file 4). Hence, the expression levels determined based on the NGS and qPCR results were compared. The expression of most analysed miRNAs showed similar trends and very high correlation coefficients, varying from 0.71 to 0.99, were found between NGS and qPCR of six miRNAs. Such high correlations were found in both roots and leaves for four miRNAs: dca-miR155 (r_roots_ = 0.83; r_leaves_ = 0.97), dca-miR266 (r_roots_ = 0.98; r_leaves_ = 0.76), dca-miR553 (r_roots_ = 0.93; r_leaves_ = 0.98) and dca-miR397b (r_roots_ = 0.99; r_leaves_ = 0.71). High correlation in roots only was found for dca-miR159a.1 (r_roots_ = 0.79) while in leaves only for dca-miR156 (r_leaves_ = 0.96). However, high correlations between NGS and qPCR results for dca-miR155 (both plant organs) and dca-miR156 (leaves) were mainly due to differential expression levels between varieties (Fig. 4).

### Expression of miRNAs related to salt stress

As indicated above, the consistency of NGS and qPCR data for some miRNAs resulted from the fact that DH1 and DLBA differed considerably in their expression but miRNA expression did not change due to salt stress. Therefore, only those miRNAs which had consistent expression patterns in NGS and qPCR were further considered.

**Figure 3:**
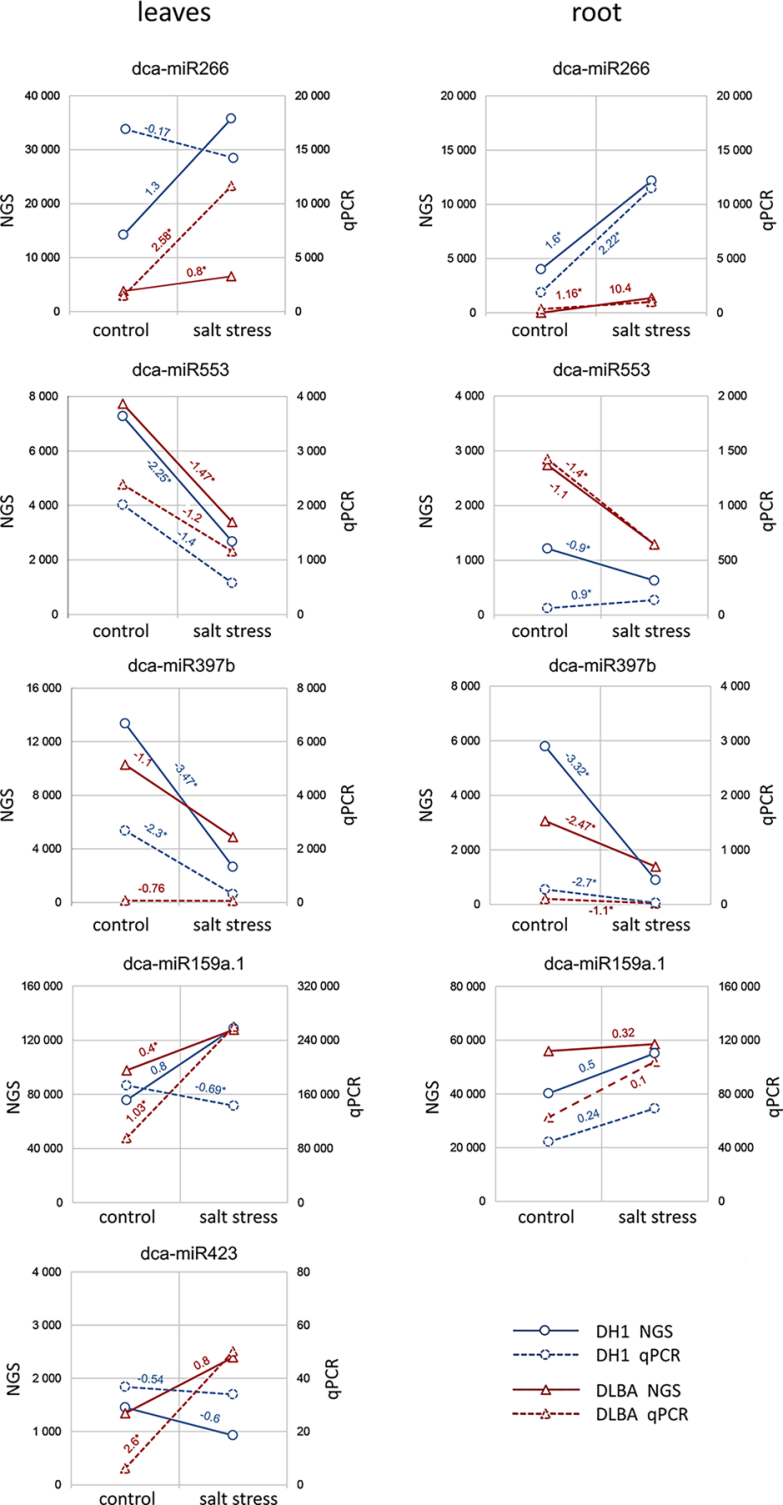
Relative expression levels of dca-miRNAs showing correlation between sRNA sequencing and qPCR results. The lines show only the direction of expression changes; expression changes are marked as log2 fold change; significant changes (*p* value < 0.05) are marked by asterisk.

When DLBA and DH1 were considered separately, five miRNAs (dca-miR266, dca-miR423, dca-miR553, dca-miR159a.1, and dca-miR397b) were identified as being potentially involved in carrot response to salt stress as their expression changed in reaction to salt treatment in a similar way in independent experiments, in at least one variety (Fig. 3). dca-miR553 was down-regulated in salt-stressed plants and its expression change was similar in leaves of both varieties (qPCR logarithmic fold change was in the range of -2.25 to -1.20, which corresponded to 4.8 to 2.3 fold decrease using a linear scale). In roots, dca-miR553 was down-regulated in DLBA while in DH1 its expression was apparently not affected by salt stress, as it was either up- or down-regulated depending on the experiment. dca-miR397b was down-regulated in roots and leaves of both varieties, but the reaction was more evident for DH1 (5–11 fold decrease) than for DLBA (2–sixfold). dca-miR266 was up-regulated in leaves and roots of DLBA while in DH1 it was up-regulated only in roots. The response in DH1 roots was more pronounced but the expression remained at much lower level than in DLBA roots. dca-miR159a.1 and dca-miR423 were up-regulated in DLBA leaves. Thus, DLBA responded to salt stress by changing expression of three miRNA in leaves and roots (dca-miR266, dca-miR397b, dca-miR553), and another two miRNAs only in leaves (dca-miR159a.1, dca-miR423). Fewer changes were found in DH1, one miRNA changed its expression in leaves and roots (dca-miR397b), another only in leaves (dca-miR553), and yet another only in roots (dca-miR266).

### Computational prediction of miRNA targets

The computational prediction of miRNA targets performed by running the psRNATarget analysis server resulted in 530 potential targets for the five miRNAs selected after qPCR validation (Supplementary file 5). Their expectation values were low and ranged between 0.5 to 5.0, and the unpaired energy (UPE) required to open the secondary structure around the target was below 25. Most of the predicted target-miRNA interactions were classified as cleavage (402 interactions) while the remaining were attributed to translational inhibition (128 interactions). The highest number of targets (136) was indicated for both dca-miR159a.1 and dca-miR423 miRNAs. The considerable number of target genes (99 genes; 18.7%) were annotated as uncharacterised. Lowering the threshold for the target expectation value to 3.5 considerably reduced the number of predicted targets to 106. Only two and eight targets were indicated for dca-miR266 and dca-miR397b, respectively, while from 25 to 45 targets were indicated for each of the remaining miRNAs. For most of these miRNAs, cleavage interaction was 1–3 times more frequent then translational inhibition while for dca-miR159a.1 and dca-miR397b cleavage interaction was 6–7 times more frequent. Values of UPE ranged from 5 to 24, and average UPE values calculated for targets of the same miRNA were similar among miRNAs (16.0 to 17.4).

### Prediction of miRNA targets based on degradome sequencing

**Figure 4:**
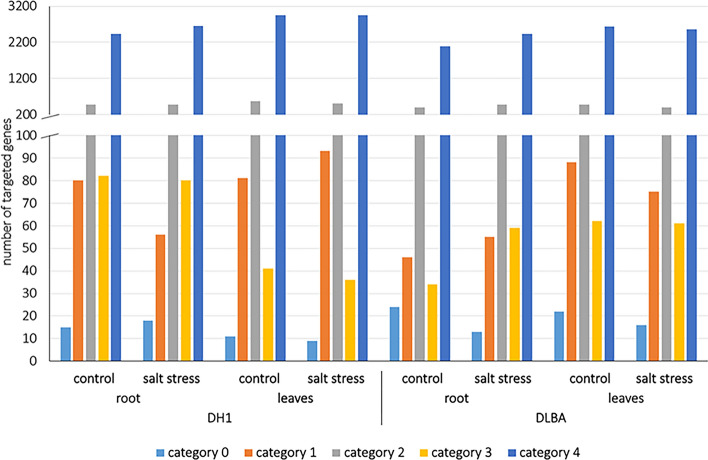
Summary of CleaveLand4 target categories for all potential miRNAs detected by degradome analyses in eight study sample types.

The second approach based on the sequencing of eight degradome libraries (in three replications each) was applied for miRNA target prediction. Sequencing data analyses indicated 25,666 miRNA targets for all miRNAs, 2,602 to 3,666 depending on the library. The targets were classified into five categories (coded as 0, 1, 2, 3, and 4) based on the frequency of fragments derived from their degradation by potential miRNAs (Fig. 4). The most relevant categories, 0 and 1, accounted for 0.5% and 2.2%, respectively, while the categories 2 and 3 accounted together for 15.6% (14.8% and 1.8%, respectively). Most target genes (80.6%) were classified to the category 4 of the lowest quality with just one degradome read at the probable cleavage position.

The degradome analysis indicated 144 targets when considering the same five miRNA as selected for computation prediction using the psRNATarget server (Supplementary file 6). The number of targets ranged from 26 to 43 for four miRNAs while only five targets were indicated for dca-miR266. Most of them (106) were classified to category 4, there were no targets in category 3, 28 targets were in category 2. In the most relevant categories, 1 and 0, there were nine and two targets, respectively (Table 1). However, two targets of dca-miR553 and dca-miR397b in category 0 were genes encoding uncharacterized proteins. In category 1, there were targets of three miRNAs: dca-miR423, dca-miR553 and dca-miR159.1. Genes of unknown function represented a significant part (29.7%) of the 144 targets.

**Table 1:** Dca-miRNA target genes predicted based on degradome data (categories 0–2).

Target function	Target	Degradome category	*p* value	miRNA name
Uncharacterized	LOC108218867	1	0.007	dca-miR159a.1
Nucleoside diphosphate kinase 1	LOC108202474	1	0.008	dca-miR159a.1
hemK methyltransferase family member 2	LOC108211733	1	0.013	dca-miR159a.1
Pentatricopeptide repeat-containing protein At1g62350	LOC108194037	2	0.021	dca-miR159a.1
Heat shock protein 83-like	LOC108227515	2	0.023	dca-miR159a.1
Ethylene-responsive transcription factor 12	LOC108193628	2	0.081	dca-miR159a.1
Uncharacterized	LOC108209986	2	0.108	dca-miR159a.1
GTP-binding protein SAR1A-like	LOC108200375	2	0.116	dca-miR159a.1
CBS domain-containing protein CBSX1, chloroplastic-like	LOC108193199	2	0.118	dca-miR159a.1
Syntaxin-121	LOC108223368	2	0.122	dca-miR159a.1
Uncharacterized	LOC108223656	2	0.141	dca-miR159a.1
Transcription factor TCP8-like	LOC108218615	2	0.004	dca-miR266
Serine/threonine-protein kinase HT1-like	LOC108227906	2	0.008	dca-miR266
Uncharacterized	LOC108214191	2	0.014	dca-miR266
Uncharacterized	LOC108220080	0	0.000	dca-miR397b
Classical arabinogalactan protein 9-like	LOC108192853	2	0.004	dca-miR397b
Uncharacterized	LOC108220890	2	0.008	dca-miR397b
Protein phloem protein 2-like A1-like	LOC108193771	2	0.009	dca-miR397b
Protein phloem protein 2-like A1-like	LOC108199724	2	0.010	dca-miR397b
Uncharacterized	LOC108200363	2	0.025	dca-miR397b
28 kDa ribonucleoprotein, chloroplastic	LOC108202773	2	0.055	dca-miR397b
Protein BPS1, chloroplastic-like	LOC108193212	2	0.068	dca-miR397b
Protein DCL, chloroplastic-like	LOC108225302	1	0.001	dca-miR423
ATPase family AAA domain-containing protein 1-B-like	LOC108214061	1	0.005	dca-miR423
Uncharacterized	LOC108211904	1	0.005	dca-miR423
Uncharacterized	LOC108197576	2	0.012	dca-miR423
17.1 kDa class II heat shock protein-like	LOC108198986	2	0.033	dca-miR423
Metal-nicotianamine transporter YSL3-like	LOC108214062	2	0.037	dca-miR423
Thioredoxin-like protein CDSP32, chloroplastic	LOC108210741	2	0.060	dca-miR423
Uncharacterized	LOC108193287	2	0.083	dca-miR423
Protein IRX15-LIKE-like	LOC108225770	2	0.098	dca-miR423
17.1 kDa class II heat shock protein-like	LOC108225374	2	0.107	dca-miR423
Uncharacterized	LOC108193364	0	0.001	dca-miR553
Histidine kinase 4-like	LOC108199538	1	0.001	dca-miR553
U-box domain-containing protein 21-like	LOC108213344	1	0.002	dca-miR553
Glutamate synthase (NADH), amyloplastic	LOC108226948	1	0.004	dca-miR553
Glucan endo-1,3-beta-glucosidase, acidic-like	LOC108211710	2	0.009	dca-miR553
Xyloglucan 6-xylosyltransferase 1-like	LOC108200535	2	0.030	dca-miR553
Splicing factor 3B subunit 6-like protein	LOC108213161	2	0.045	dca-miR553

The list of all targets is in Supplementary File 5. For detailed information about degradome categories based on the CleaveLand4 pipeline^24^.

A comparison of results obtained using psRNATarget (Supplementary file 5) and degradome sequencing (Supplementary file 5) revealed 17 targets being outputs from both approaches (marked in Supplementary file 6). They constituted only 3.2% and 11.8% of targets indicated by psRNATarget analysis and degradome sequencing, respectively. Fourteen of the 17 targets have been annotated but only seven of those had GO terms assigned.

### Pathway and GO term enrichment analysis

**Figure 5:**
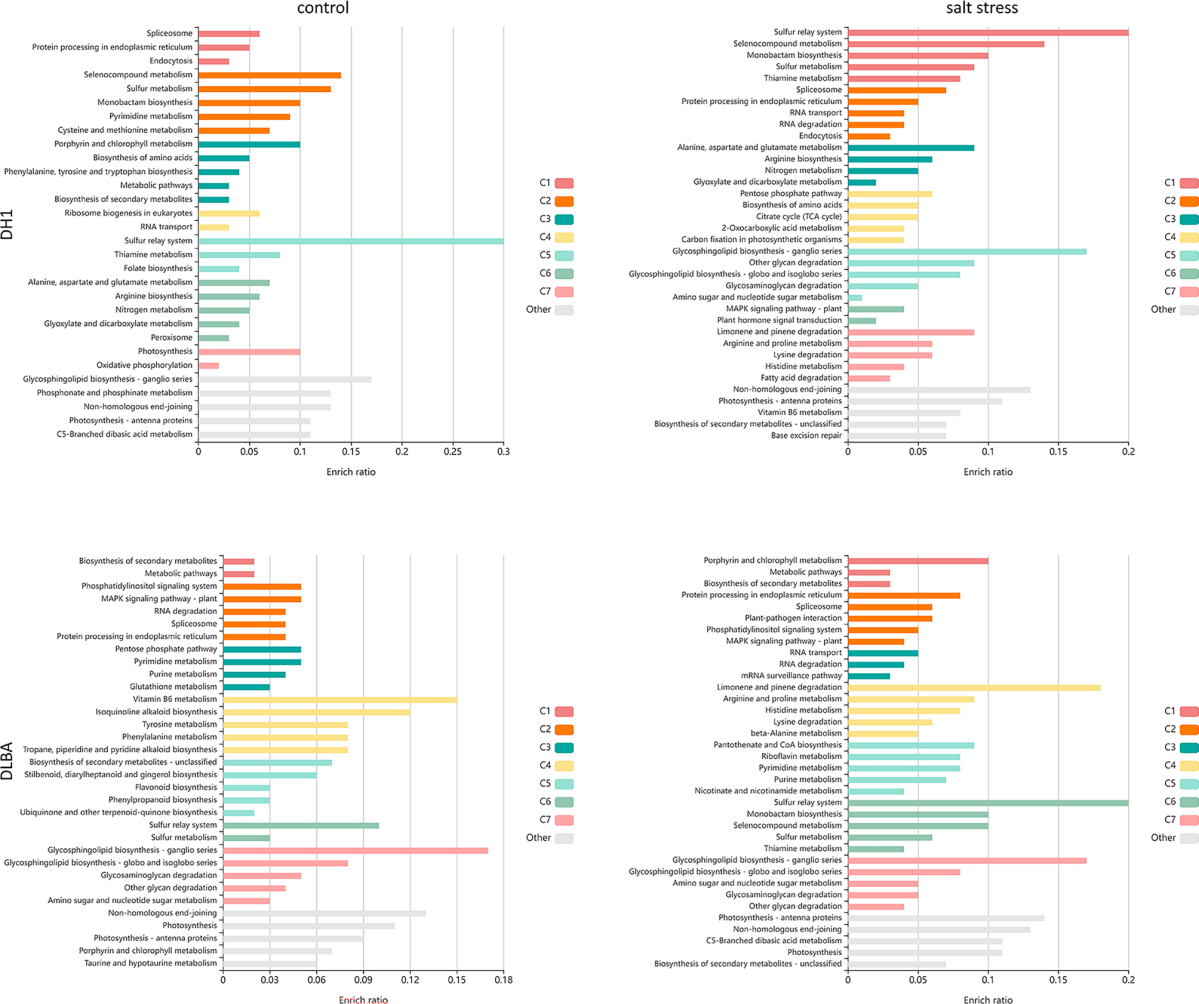
Exploratory visualization of the enriched results from pathway analysis by KOBAS. Bars represent *p* values for terms in different clusters consisting of colour mega-cluster (from C1 to C7) and five top clusters not matched to any mega-cluster (light grey bars).

Pathway enrichment analysis using KOBAS was performed for carrot miRNA target genes. The results for DH1 showed that genes selected based on the results of degradome sequencing mapped to 96 and 95 pathways for the control and salt-stressed plants, respectively. For DLBA, genes matched to 81 and 97 pathways, respectively. The number of significantly enriched pathways varied depending on the carrot variety and treatment. Eight pathways were indicated for the salt-stressed DH1 carrot and that number was lower by 38% than for the control DH1 plants (13 pathways) (Table 2). Among them, only one pathway was uniquely enriched in the salt-stressed plants, e.g., the ‘Alanine, aspartate and glutamate metabolism’ pathway. The other seven enriched pathways were assigned to both the control and salt-stressed plants. The reaction of DLBA plants was different, 14 pathways were enriched, including 13 pathways in the salt-stressed plants of which five were identified for that treatment in DLBA plants only (‘RNA transport’, ‘Purine metabolism’, ‘Arginine and proline metabolism’, ‘Amino sugar and nucleotide sugar metabolism’, and ‘Biosynthesis of secondary metabolites’). In the control DLBA plants, four pathways were enriched and one of them was unique (‘MAPK signalling pathway’). Hence, six pathways were identified that were enriched only in DLBA and three only in DH1, the above mentioned ‘Alanine, aspartate and glutamate metabolism’ pathway, and also ‘Sulfur relay system’ and ‘Sulfur metabolism’ (Fig. 5). Only six target genes confirmed by qPCR miRNAs were identified in these pathways and they were often assigned to more than one pathway (Supplementary file 7). They were targets of three miRNAs, i.e., dca-miR159a.1, dca-miR423 and dca-miR553.

**Table 2:** Summary of KEGG enrichment analysis of target genes for all miRNA candidates.

Pathways	DH1		DLBA	
	Control	Salt	Control	Salt
Number of input genes	437	359	340	412
Number of mapped metabolic pathways	96	95	81	97
Number of statistic significant pathways	13	8	4	13
Alanine, aspartate and glutamate metabolism		+		
Amino sugar and nucleotide sugar metabolism				+
Arginine and proline metabolism				+
Biosynthesis of amino acids	+	+		
Biosynthesis of secondary metabolites				+
Cysteine and methionine metabolism	+	+		
MAPK signalling pathway—plant			+	
Metabolic pathways	+	+	+	+
Photosynthesis	+		+	+
Photosynthesis—antenna proteins	+	+		+
Plant-pathogen interaction	+			+
Porphyrin and chlorophyll metabolism	+			+
Protein processing in endoplasmic reticulum	+	+		+
Purine metabolism				+
Pyrimidine metabolism	+			+
Ribosome	+	+		
RNA transport				+
Spliceosome	+	+	+	+
Sulphur metabolism	+			
Sulphur relay system	+			

KOBAS was used for the identification of biochemical pathways that are significantly enriched (adjusted p-value < 0.05, marked with plus ‘+’) in genes that were found to be potentially regulated by dca-miRNA.

The GO terms enrichment analysis using KOBAS did not confer significant results hence, the DAVID tool involving a wider range of databases was used to analyse target genes of the selected five miRNAs. Out of 674 genes, 200 were annotated to functional terms and used for clustering. They were attributed to 15 clusters, depending on functional terms (from 3 to 15 terms per cluster), where a single term was represented by 3 to 69 genes (median = 6, mean = 11) (Supplementary file 8). Fourteen functional terms were significantly enriched jointly in the first five clusters. The percentage share of individual miRNAs was assessed in clusters with significant representations of miRNA target genes per enrichment term. The target genes for dca-miR423 were mostly represented in cluster 1 (45.1%) and cluster 2 (37.8%) assigned to two terms, i.e. ‘helicases from superfamily 1/2 with ATP-binding domain’ and ’protein serine/threonine kinase activity’, respectively. Targets of three miRNAs (dca-miR553—30.8%, dca-miR423—27.0%, dca-miR159a.1—21.9%) had a similar contribution to cluster 3 assigned to the ‘integral components of membrane’ term. Cluster 4 contained 39.1% targets for dca-miR397b, and was assigned to the ‘proteins with tetratricopeptide repeat’ term. Cluster 5, assigned to the ‘F-box domain, cyclin-like’ term included targets mostly for dca-miR266 (70.6%).

### Expression of miRNA target genes

**Figure 6:**
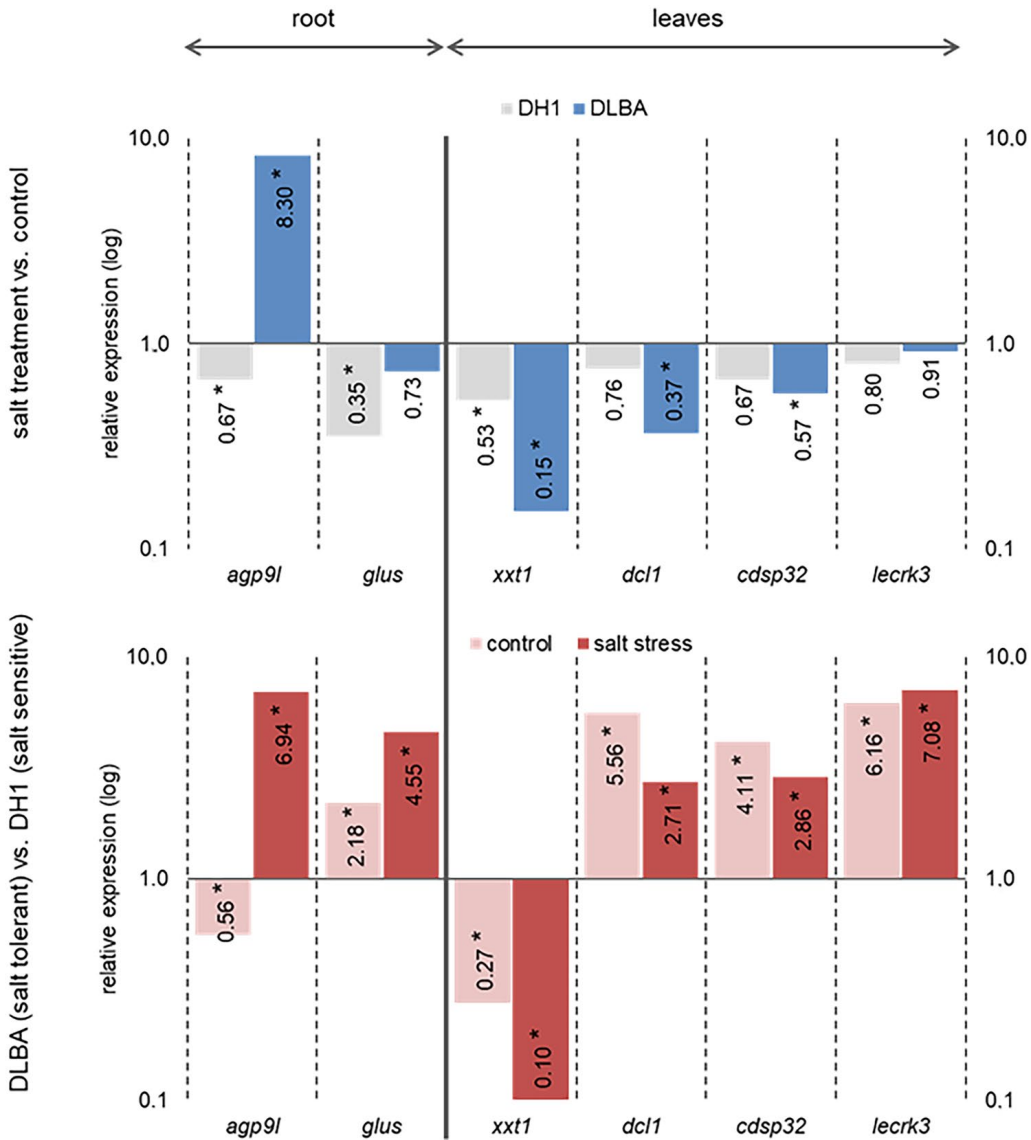
Relative expression of selected miRNA target genes obtained by the comparison of salt stress to control conditions (upper row) and DLBA to DH1 (bottom row). Gene abbreviations: *agp9l*—arabinogalactan protein 9-like; *cdsp32*—chloroplastic drought-induced stress protein of 32 kD; *dcl1*—defective chloroplasts and leaves protein 1; *glus*—glutamate synthase; *lecrk3*—lectin S-receptor-like serine/threonine-protein kinase 3; *xxt1*—xyloglucan 6-xylosyltransferase 1-like. Differential expression marked by asterisk (*), significant at *p* = 0.05.

Expression of selected target genes was verified by qPCR and then relative expressions were assessed by comparing expression levels in: 1) the salt-stressed plants vs. control for DLBA and DH1 varieties, and 2) in DLBA vs. DH1 for the control and salt-stress treatments (Fig. 6). In the roots, differential expression was found between varieties. The arabinogalactan protein 9-like (*agp9l*) expression was highly up-regulated in the salt-stressed DLBA root while down-regulated in DH1. The glutamate synthase (*glus*) expression did not change in DLBA but was down-regulated in DH1. The expression of both genes, *agp9l* and *glus*, was much higher (5 and 7 times, respectively) in the salt-stressed DLBA plants than in DH. In the leaves, the salt stress down-regulated the expression of xyloglucan 6-xylosyltransferase 1-like (*xxt1*) gene that was more evident in DLBA. This variety had also a lower *xxt1* expression than DH1 in the control and salt stress conditions. Two genes, defective chloroplasts and leaves protein 1-like (*dcl1*) and chloroplastic drought-induced stress protein (*cdsp32*), were down-regulated in DLBA and not in DH1, and the lectin S-receptor-like serine/threonine-protein kinase 3 (*lecrk3*) gene expression did not change in the plants exposed to the salt stress. Despite that, all three genes were 3–7 times more up-regulated in the DLBA than in DH1 plants, both in the control and salt stress conditions.

## Discussion

In the present study, we have investigated a potential role of miRNAs as mediators in carrot tolerance to salt stress. For that purpose, small RNA libraries obtained from the leaf and root samples of salt-tolerant DLBA and salt-sensitive DH1 plants, as shown earlier^10^, grown in the non-saline (control) or saline soil were sequenced, and miRNAs were identified. The number of obtained sRNA reads was comparable for DH1 and DLBA varieties (33.8M and 35.4M, respectively), and higher than that reported previously for carrot (18.9 M to 25.5 M reads depending on variety)^23^. The obtained results confirm a general consensus that 21–24 nt reads are the most abundant, with the 24 nt fraction being most numerous in both varieties^25^. The precise excision of mature miRNA from its precursor is a critical aspect of miRNA biogenesis. . The length of pre-miRNAs varies usually from 50 to 900 nt (mean = 146 nt), but a fraction of miRNA shorter than 336 nt constitutes 98% of characterised precursors^26^. Our results are highly congruent with these data. All identified pre-miRNA were shorter than 245 nt, and majority of pre-miRNA (75%) were shorter than 186 nt. Based on the aforementioned criteria, and taking into account that most plant miRNAs are 21 or 22 nt-long, and a 21 nt long molecule with 5′-uridine is characteristic of DCL1 cleavage and AGO1 association, ten candidate miRNAs were selected for validation using RT-qPCR, with regard to their expression patterns in carrot plants, which is required for definitive miRNA recognition as suggested previously^27^. Two independent experiments were conducted in two consecutive years. Therefore, the growth of carrot plants had been affected by varying conditions during vegetation, except for the same salt stress treatment. The selected miRNAs were expressed in DLBA and DH1 plants in both experiments as determined by NGS and qPCR in the first and the second year, respectively. Hence, the results provided experimental confirmation for the presence of miRNAs.

The initial identification of miRNAs in carrot, completely based on *in-silico* screening of conserved across plant species pre-miRNA sequences in the carrot Expressed Sequence Tags (EST) database indicated only 17 miRNAs^28^. Then, using a sequencing approach, 144 conserved and 99 novel miRNAs were identified in two colour variants of carrots^23^. Our study led to identification of 24 plant conserved miRNA, including those identified by Bhan et al.^23^ such as miR166, miR159, miR171_1, miR156, miR396, miR164 and miR167, and additionally, further 71 novel carrot miRNAs. Methodological differences might lead to identification of different miRNA and their number. In the present study, criteria of miRNA identification were more restrictive e.g., reads threshold > 300 instead of > 10 and exclusion of sequences longer than 25 nt. It has been also widely accepted that miRNA expression is specific to variety, organ, tissue, plant developmental stage or stress treatment^29,30^, and these factors were applicable in the previous and our studies. More conserved and novel miRNA were identified for DH1 than DLBA, and this relation was maintained in response to salt stress indicating that miRNA controlled regulation was more active in the salt-sensitive variety, at least in fully developed plants after a long-term growth in the saline soil.

Available reports concerning 41 plant species exposed to elevated NaCl concentrations (80–600 mM) have indicated that the role of miRNA is multifaceted and different in tolerant and sensitive varieties^31^. For example, a higher expression level of ghr-miR397b was observed in a salt-tolerant cotton cultivar. The expression of another miRNA, ghr-miR159, did not change in the salt-tolerant variety after NaCl treatment, while it decreased more than three times in a salt-sensitive cultivar^30^. A counteracting activity of the same miRNA was also shown. In wheat, miR159 was down-regulated after 150 mM NaCl treatment^32^ while it was up-regulated after 200 mM NaCl treatment in another study^33^. Moreover, miRNA interactions are complex as one miRNA may bind to several target transcripts and can be involved in regulation of different processes. Also several miRNAs can activate or repress mechanisms in the same pathway^34^. Analogously, in the present work, similar responses manifested by miRNA differential expression between DH1 and DLBA varieties, in leaves and roots, were observed in the control and salt-stressed plants. Only eight miRNA candidates were expressed in both varieties, regardless of the plant organ and treatment. A variety-specific response was evident, for example, in case of dca-miR397b expression which decreased in salt-stressed plants of both varieties, but the decrease was more pronounced in DH1. An opposite response was exemplified by down-regulation of a novel dca-miR553 in the roots of DLBA but not in DH1.

The comparison of miRNA expression in two independent experiments followed by qPCR validation indicated five miRNAs, three in the root (dca-miR397b, dca-miR159a.1, dca-miR266) and three in the leaves (dca-miR397b, dca-miR553, dca-miR423), which may play a considerable role in carrot plant response to salt stress. Two of those, miR397b and miR159a.1, are conserved and were identified earlier across many plant species, including important model plants, such as rice^35^ and Arabidopsis^36^, while the other three (dca-miR266, dca-miR423 and dca-miR553) are carrot-specific miRNAs. The expression of dca-miR159a.1 was very high (75–127 and 40–58 thousand reads in the leaves and roots, respectively), much exceeded the expression of other miRNAs, and was constitutively higher in the DLBA control plants than in DH1.

**Figure 7:**
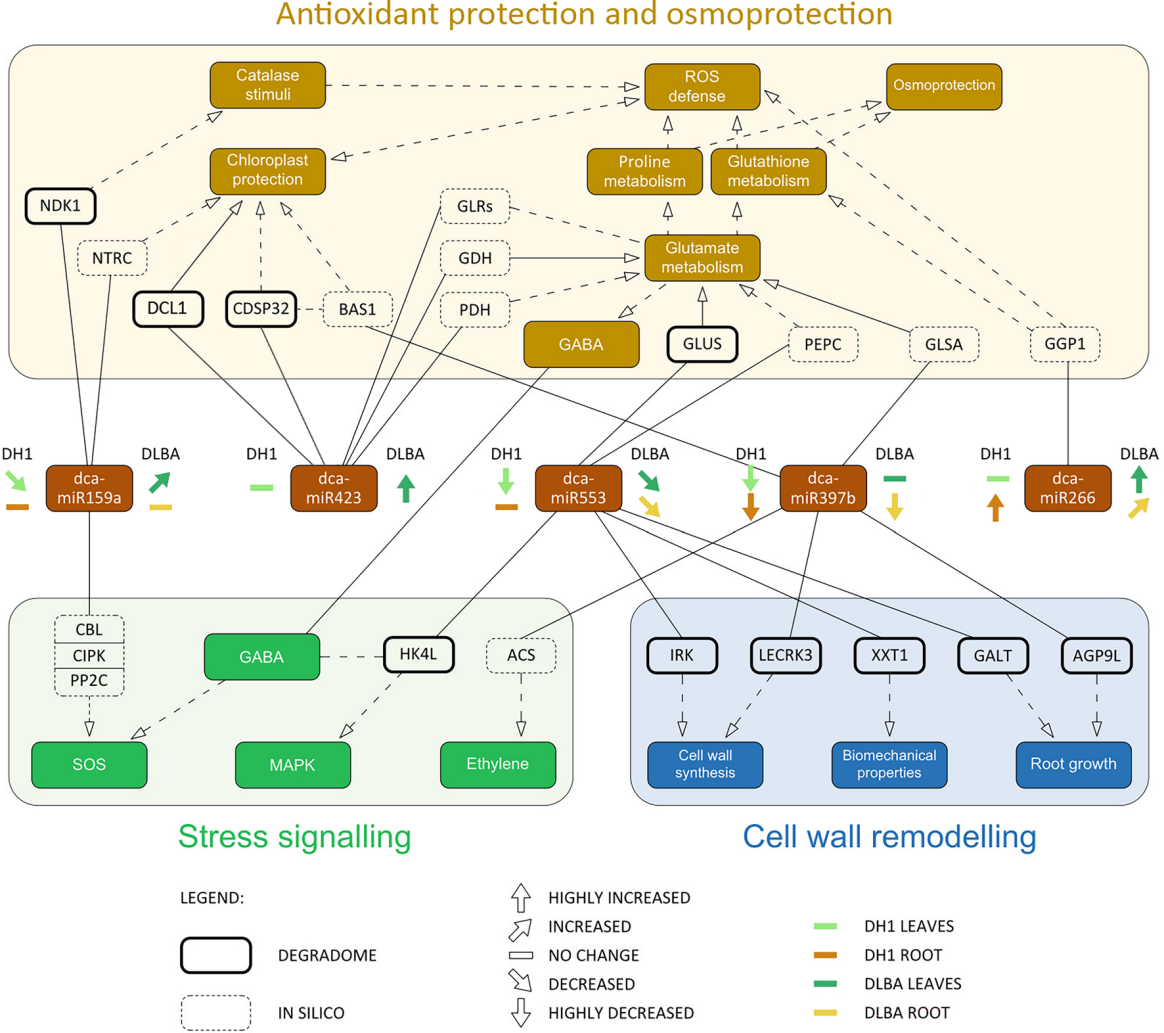
MicroRNA with their target genes potentially involved in carrot response to salt stress. Lines and dashed lines show, respectively, directed and downstream interactions between miRNAs, target genes and cellular processes. ACS—1-aminocyclopropane-1-carboxylate synthase; AGP9L—arabinogalactan protein 9-like; BAS1—phyB activation-tagged suppressor 1; CBL—calcineurin B-like; CDSP32—chloroplastic drought-induced stress protein of 32 kD; CIPK—CBL-interacting protein kinases; DCL1—defective chloroplasts and leaves protein 1; GABA—gamma-aminobutyric acid; GALT—hydroxyproline-*O*-galactosyltransferase; GDH—glutamate dehydrogenase; GGP1—gamma-glutamyl peptidase 1; GLRs—glutamate receptors; GLSA—glutaminase; GLUS—glutamate synthase; HK4L—histidine kinase 4-like; IRK—probable LRR receptor-like serine/threonine-protein kinase; LECRK3—lectin S-receptor-like serine/threonine-protein kinase 3; MAPK—mitogen-activated protein kinase; NDK1—nucleoside diphosphate kinase 1; NTRC—NADPH-thioredoxin reductase, type C; PP2C—protein phosphatase 2C-type; PDH—pyruvate dehydrogenase; PEPC—phosphoenolpyruvate carboxylase; SOS—Salt Overly Sensitive pathways; XXT1—xyloglucan 6-xylosyltransferase 1-like gene.

Prediction of miRNA targets in carrot combined with further GO and KEGG enrichment analyses revealed that the identified miRNAs may have diverse roles and are potentially involved in mechanisms assigned to particular pathways contributing to salt stress response. Several targets were predicted for each miRNA, thus one miRNA could participate in different biological processes. Among them, osmoprotection and reactive oxygen species (ROS) scavenging are distinguished and linked with hormonal signalling. Further, these miRNAs were also involved in cell wall remodelling and signal transduction pathways (Fig. 7).

Experimental validation of the predicted targets can be done by degradome analysis designed to identify cleavage sites^37,38^. The KEGG enrichment of miRNAs target genes identified by degradome sequencing revealed differences in degradation rates between DH1 and DLBA indicating differential miRNA activities. Only one pathway was uniquely enriched in the salt-stressed plants of salt-sensitive DH1, e.g., the ‘Alanine, aspartate and glutamate metabolism’ pathway while five pathways were identified uniquely in the tolerant DLBA variety (‘RNA transport’, ‘Purine metabolism’, ‘Arginine and proline metabolism’, ‘Amino sugar and nucleotide sugar metabolism’, and ‘Biosynthesis of secondary metabolites’) (Table 2).

Salt stress usually modifies the content and profile of free amino acids (FAA) and their derivatives. The osmolytes, such as proline, glycine, and glycine betaine support the maintenance of intracellular osmotic potential under saline conditions. The changes in osmolyte contents were previously reported in the salt-stressed DLBA and DH1 plants. The proline content increased up to sevenfold in DLBA while in DH1 the increase was only about twofold. The proline contribution to the total amount of FAA was also higher in DLBA (26.1%) than in DH1 (3%) after NaCl treatment^9^. Thus, both varieties showed an enhanced proline accumulation, but this process was much more efficient in DLBA, particularly in roots. Proline is synthesized from glutamate (Glu) via the glutamate semialdehyde and pyroline-5-carboxylate (P5C) pathway or via the arginine-ornithine-P5C pathway. The over-representation of genes associated with metabolic pathways identified based on the degradome data suggests that their transcripts may undergo increased regulatory processing during salt stress. The enrichment analysis revealed that genes related to the arginine and proline metabolism were over-represented in the degradome of the salt-treated DLBA, but not DH1 plants. The activity in this pathway distinguished salt-stressed DLBA plants from the control, but also from the DH1 plants. For the latter, another pathway, the ‘Alanine, aspartate and glutamate metabolism’ pathway, was identified as significantly represented by potential miRNA targets in salt-stressed plants. These two pathways led to diverse metabolites, but they are related to nitrogen metabolism, and Glu is a common precursor for both of them. The analyses of miRNA targets indicated three miRNAs, two novel (dca-miR533 and dca-miR423) and one conserved (dca-miR397b), targeting three Glu related genes: glutamate synthase (*glus*), glutamate dehydrogenase (*gdh*) and glutaminase (Fig. 7). The *glus* gene was the target for dca-miR553. The expression of dca-miR553 decreased in the salt-stressed plants that may led to up-regulation of Glu biosynthesis genes. Such decrease of dca-miR533 expression was evident in the roots of salt-tolerant DLBA and not in salt-sensitive DH1. Also dca-miR397b targeting the glutaminase gene was down-regulated in the roots and leaves of salt-stressed DLBA and DH1 plants. Glutaminase removes an amino group from glutamine, which led to Glu biosynthesis. Reversely, Glu can be converted to 2-oxoglutarate by GDH. The *gdh* gene was found to be the target for dca-miR423, which expression was up-regulated in the leaves of salt-stressed DLBA plants. Thus dca-miR423 up-regulation can limit Glu metabolism and support its higher level. The dca-miR553 and dca-miR423 target also gene transcripts related to the citrate cycle (TCA), in which citrate is converted to 2-oxoglutarate after several reactions and eventually led to Glu biosynthesis. Although, the up-regulation of dca-miR423 targeting pyruvate dehydrogenase may limit the supply of substrates for citrate biosynthesis, the down-regulation of dca-miR553 may up-regulate phosphoenolpyruvate carboxylase and activate an alternative, shorter pathway branch for citrate biosynthesis through oxaloacetate. Furthermore, glutamine is a precursor for purine metabolism, and the purine metabolism pathway was another pathway identified only in the salt-stressed DLBA plants in the enrichment analysis. Hence, these results strongly indicate that the Glu biosynthesis and metabolism may undergo enhanced miRNA control in carrot exposed to salt stress, and these processes are more pronounced in the salt-tolerant plants. Target gene expression analysis confirmed that the expression of *glus* gene was much higher in the roots of salt-tolerant DLBA plants than in the salt-sensitive DH1, and such relation was observed in plants exposed to the salt stress but also in the control. The salt stress treatment repressed *glus* expression in DH1, unlike in DLBA. These results are congruent with previous results concerning osmolate content and indicate that the biosynthesis of Glu-derived compounds may be less efficient in the salt-sensitive DH1 plants due to a lower activity of the Glu biosynthesis while in the salt-tolerant DLBA plants, the supply of Glu remains unchanged.

Glu metabolism affects the availability of proline and glutathione (GSH) and thus the activity of antioxidant mechanisms, too. Increased salt concentrations lead to H_2_O_2_ accumulation and production of ROS^39^. Salinity-induced ROS scavenging pathways in chloroplasts are based on tightly coordinated processes related to the water–water cycle, stromal ascorbate–glutathione (AsA–GSH/GSSG) cycle, thioredoxin-peroxiredoxin (Trx–Prx) system, and non-enzymatic scavenging system^40^. Also proline participates in scavenging free radicals and contributes to NADP + /NADPH homeostasis^41^. Among potential miRNA targets involved in antioxidant protection, a gene coding for gamma-glutamyl peptidase 1 (GGP1), which degrades GSH, was identified. Thus, the increased expression of dca-miR266 observed in the leaves of tolerant DLBA plants under salt stress could provide a higher GSH level and its availability in the GSH-GSSG cycle. The aforementioned results are congruent with previous findings indicating that more GSH was oxidized and a regenerative system of GSH was more efficient in the salt-tolerant DLBA plants^9^. In consequence, the balance in AsA-GSH-NADPH cycle required for efficient photosynthesis in chloroplasts could be maintained^42^. Another two genes coding defective chloroplasts and leaves protein 1 (DCL1) and a probable thiol-disulfide oxidoreductase *CDSP32* (LOC108210741), a chloroplastic gene encoding thioredoxin-like protein, were identified as the dca-miR423 targets. qPCR analysis showed that their expression was down-regulated in the salt-stressed plants, but the salt-tolerant DLBA plants maintained the expression of both genes at significantly higher levels than salt-sensitive DH1, regardless of whether salt stress was applied or not, hence chloroplast protection mechanisms seem to be more efficient in DLBA. DCL1 is a plastid-localized protein involved in correct chloroplast and palisade cells development in tomato leaves^43^. CDSP32 interacts with 2-Cys peroxiredoxin BAS1 (the dca-miR397b target; LOC108224024) and protects the photosynthetic apparatus against oxidative damage during leaf development in *Arabidopsis*^44^. BAS1 was up-regulated in barley and wheat during drought^45^ and salt stress^46^, respectively. Thus, different ability for biomass production and efficiencies of antioxidant protection mechanisms in DLBA and DH1 found previously^9^ could be further due to a simultaneous activity of dca-miR397b and dca-miR423. Furthermore, the nucleoside diphosphate kinase 1 (NDK1; LOC108202474) was identified as the dca-miR159a.1 target. NDK1 interacts with catalase (CAT), and plants over-expressing NDK1 have an increased ability to eliminate exogenous H_2_O_2_^47^. Differential expression of dca-miR159a.1 corresponded to changes of CAT activities in the DLBA and DH1 leaves after salt treatment^9^.

Cell wall remodelling occurs during plant response to environmental changes. arabinogalactan proteins (AGPs) present in the wall of various cell types play role in plant growth and developmental processes, xylem differentiation and signalling pathways^48^, and their increased levels are considered to be the result of plant adaptation to salinity^49^. In saline conditions, the cell wall polysaccharide components such as cellulose, hemicellulose, lignins, pectins, and callose are subjected to compositional and structural modifications^50^. Genes coding for proteins involved in cell wall remodelling were identified as targets of dca-miR397b (classical arabinogalactan protein 9-like protein AGP9L) and dca-miR553 (xyloglucan 6-xylosyltransferase 1-like XXT1 and hydroxyproline-*O*-galactosyltransferase GALT, which catalyses AGP *O*-glycosylation). The target gene expression analysis confirmed that the expression level of *agp9l* highly increased in response to salt stress in salt-tolerant DLBA while it decreased in salt-sensitive DH1. In contrast, the expression of *xxt1* gene was repressed in both varieties but more so in DLBA, so greater changes in the cell wall composition are expected in salt-tolerant DLBA. Inversely correlated expression of *agp* and *xxt* genes was also reported earlier in Arabidopsis^51^. Other target genes related to processes associated to the cell wall seem to be regulated by miRNA in the tolerant variety exposed to salt stress. Fasciclin-like AGP has a similarity to SALT-OVERLAY SENSITIVE 5 (SOS5/FLA4) being most likely involved in sensing turgor pressure, cell wall integrity, and signal transduction to the cell wall leucine-rich repeat (LRR) receptor-like kinases (RLKs) system to stimulate the expression of cellulose synthase and regulate cell wall synthesis^52^. The cluster of 36 protein kinases related genes was significantly represented in the list of carrot miRNA targets and three RLK genes (LOC108197357, LOC108201804, and LOC108209279) found in the degradome data may contribute to the aforementioned processes. A probable LRR receptor-like serine/threonine-protein kinase IRK has been proposed to be a root cell division controller^52^. Differential expression of dca-miR553 targeting the IRK gene supports higher activity of cell wall synthesis in the DLBA roots and is congruent with better growth of this tolerant variety in saline soil^10^. Another RLK, the G-type lectin S-receptor-like serine/threonine protein kinase LECRK3, the target of dca-miR397b, encodes a receptor-like kinase with serine/threonine kinase activity whose expression was induced by severe salt stress^53^, and Arabidopsis RLK overexpressors showed improved salt tolerance and higher yields under salt stress^54^. We found that *lecrk*3 expression was not affected by salt stress, but the tolerant variety had several times higher *lecrk*3 expression under both control and salt stress conditions. Signal transductors and signalling pathways have been reported as participating in plant response to salt-stress, including GABA, ethylene, SOS and MAPK pathways. The histidine kinase 4 (HK4) is a cytokinin receptor transmitting the stress signal to a downstream MAPK cascade and it acts as a regulator of drought and salt stress responses^55^. The *HK4-like* (*DcHK4* or *DcCRE1*; LOC108199538) gene was a target of dca-miR553, which expression was reduced in the tolerant variety under salt stress.

Other probable ethylene signalling and SOS pathway mediators were indicated and further extended the list of potential regulatory factors. Among them, six genes containing the myb domain, including *MYB32* TF (LOC108202253) and two *GAMYB-like* TFs (LOC108222697 and LOC108210186) undergoing miRNA-mediated cleavage were identified. *MYB32* is induced by NaCl and drought stress, and in response to signalling molecules such as ethylene, JA, SA, ABA and cytokinins. The MYB32 TF participates in the regulation of biosynthesis of secondary wall components acting as a repressor of lignin biosynthesis, thus its activity may favour the redirection of the cell wall saccharides biosynthesis towards xylan or cellulose, hence cell wall remodelling^56^. Moreover, MYB32 interacts with the PLATZ2 TF involved in the regulation of the SOS signalling pathway^57^. GAMYB-like transcription factors (TFs) are involved in the biosynthesis and metabolism of cell wall components^58^. In *Arabidopsis*, regulation of GAMyb TFs was achieved by over-expression of miR159 providing evidence of a modulatory function of this miRNA, and which expression is salinity-induced^59^. The identification of these TF genes as dca-miR159a.1 targets in carrot is consisted with available reports showing miR159 is present in the majority of land plants where it regulates TF expression reviewed by Millar et al.^59^. Further, a differential expressions of dca-miR159a.1 and dca-miR553 in DLBA and DH1 point at regulatory differences in the salt-sensitive and salt-tolerant varieties.

## Conclusions

The NGS of small RNAs led to identification of novel miRNAs in the salt-sensitive and salt-tolerant carrot varieties that considerably enriched a pool of known carrot miRNA. The expression of miRNA changed as a result of plant exposure to salt stress. Two conserved miRNA related to salt stress response in various plant species (dca-miR397b and dca-miR159a.1) were identified in these varieties and another three novel carrot miRNAs were found. Among them, dca-miR553 showed multifaceted roles potentially targeting genes involved in various mechanisms of plant response to stress, namely cell wall remodelling, antioxidant protection and osmoprotection, and signal transduction, with simultaneous contribution of another novel dca-miR423 and conserved dca-miR397b. Differential expression of dca-miR553 between the salt-sensitive and salt-tolerant varieties was particularly manifested by its down-regulation in the roots of tolerant plants. This suggests higher activity of targets presumably enhancing efficiencies of processes such as glutamate biosynthesis and metabolism, a key factor influencing accumulation of osmoprotectants and activity of antioxidant mechanisms, including those involving glutathione. Thus the obtained results of miRNA expression, supported by the detection of differential expression of selected target genes, are congruent with previous findings showing adjustment of physiological processes in the tolerant carrot variety to growth in the saline soil^9^, but the role of putative targets requires further verification by stimulating their overexpression and silencing. Furthermore, the obtained results indicate that changes in miRNA profiles were often greater in the sensitive than in the tolerant variety, and that the expression of some miRNA targets was higher in the tolerant variety, even under control conditions. This is consistent with previous conclusions^9,10^ that the tolerant variety has been adapted to growth under saline conditions and is therefore less sensitive to high salinity.

## Material and methods

### Plant materials and treatment

Two carrot (*Daucus carota* L. ssp. *sativus* Hoffm.) varieties were used: a salt-sensitive doubled haploid (DH1) line derived from the Nantes western carrot type, and a salt-tolerant DLBA variety of the eastern type originating from cultivation area of highly saline soil in Iran. Plants were subjected to salt stress treatment as described previously^10^. In brief, plants were growing in plastic containers filled up with soil of EC = 0.2 dS·m^-1^ (control) or EC = 3.0 dS·m^-1^ (salt stress) due to irrigation with 100 mM NaCl solution prior and during vegetation. Root and leaf samples were collected from 5 to 8 individual, 14-week-old plants, from each of three independent replications. Root sampling was done by cutting off a disc from the central part of developed storage root; leaf sampling was done by collecting blades of 3–4 leaves. Samples were immediately frozen in liquid nitrogen and then used for sRNA and degradome sequencing. Analogous experiment and plant treatment was repeated in the next year, and the root and leaf samples were collected for expression analysis using quantitative polymerase chain reaction (qPCR).

Seeds (DH1 variety) were obtained from prof. P.W. Simon (Univ. of Wisconsin-Madison and USDA-ARS, Madison, WI, USA), and from the Univ. of Agriculture in Krakow carrot collection (DLBA variety, accession id: DLBA). The study complies local, Polish and international regulations.

### RNA isolation

The total RNA isolation was conducted using TRI Reagent (Zymo Research) and purified using the Direct-zol™ RNA MiniPrep Plus (Zymo Research) followed by DNA elimination using Turbo DNA-free™ Kit (Invitrogen, Thermo Fisher Scientific) according to the manufacturer’s instructions. The same RNA isolation workflow was applied for preparing sRNA and degradome libraries and samples for qPCR analysis.

### Small RNA library construction and sequencing

The RNA quality for library construction was checked with the 2100 Bioanalyzer, and qualified libraries were amplified (Illumina) and sequenced single end on the HiSeq2000 System (Illumina) by third parties (BGI, Shenzhen, China). Sequencing coverage was 10 million reads of 18–30 base in length for each sample. To identify the potential miRNAs and their targets, 24 libraries (2 varieties × 2 plant parts × 2 treatments × 3 reps) for miRNA and degradome sequencing were constructed.

### Identification and expression analysis of carrot miRNAs

Quality of raw sRNA sequencing data were checked utilising the FastQC tool. Bioinformatic analysis was performed using MTide^61^ package combining algorithms of computational tools. At first, the adaptor sequences (Cutadapt) and sequences outside the 18–30 nt length range (CleanReads.pl) were removed. Then filtered reads were mapped to other non-coding RNA (filter.pl) and to the reference genome (map_parse.pl) (reference genome version: ASM162521v2). Subsequently, novel and known miRNA were identified (miRDeep2.pl).

### Degradome library sequencing and analysis

Total RNA was extracted as mentioned above and mRNAs with 5′-monophosphates were ligated to the 5′ adaptor, followed by a reverse transcription and PCR. Degradome sequencing was performed on the IlluminaHiSeq system. Sequencing was done by third parties (BGI, Shenzhen, China). After adaptor removing, raw sequencing data were used for further bioinformatic analysis with the MTide second module based on a modified CleaveLand4.pl pipeline to identify target cleaved sites. The results were assigned to five categories (from 0 to 4) with calculated *p*-values^62^. Based on the obtained data, a modified TAPIR^63^, an integral part of MTide pipeline, was used for miRNA target prediction.

### The miRNAs target prediction

The prediction of carrot miRNA targets was done using the psRNATarget server (http://www.zhaolab.org/psRNATarget)^64,65^. The psRNATarget algorithm scans for potential targets for specified miRNA sequences based on default settings, i.e., the maximum expectation set to 5.0, and the target accessibility-maximum energy to unpair the target site (max UPE) set to 25.

### Quantitative RT-PCR analysis of miRNAs

qRT-PCR analyses based on TaqMan™ Small RNA Assays (Applied Biosystems, Waltham, MA, USA)^66^ were used to quantify miRNAs, which were previously indicated based on bioinformatic analysis. A stem-looped primer for reverse transcription and a miRNA cDNA sequence-specific assay (FAM labelled) was used for monitoring and quantifying real-time PCR (Supplementary file 9). The procedure was conducted according to the TaqMan™Small RNA Assays User Guide (Publication Number 4364031, Thermo Fisher Scientific, Waltham, MA, USA; TaqMan™Small RNA Assays User Guide)^66^ using the QuantStudio 3 Real-Time PCR System (The Applied Biosystems). The dca-miR307 miRNA, stably expressed across all objects as determined based on RNASeq data and qPCR analysis, was chosen as the internal control. A miRNA expression analysis was performed using the Rest2009 software^67^, Relative Expression Software Tool http://rest.gene-quantification.info).

### Quantitative RT-PCR analysis of target genes

Real-time qPCR was carried out with the Maxima SYBR Green/ROX qPCR Master Mix (Thermo Fisher Scientific) in three technical replicates. Reactions were prepared in 15 μL volumes consisting of 7.5 μL SYBR, 0.6 μL forward and 0.6 μL reverse 5 μM primers (sequences listed in Supplementary File 10), and 3 μL cDNA template. The thermal cycling conditions of the QuantStudioTM 3 System (Applied Biosystems, Foster City, CA, USA) were as follows: 95 °C for 10 min, 40 cycles of 95 °C for 10 s and 60 °C for 45 s. The amplification procedure included a single product verification in a melt curve analysis. The expression data was normalized using the actin-7 gene, and at least three biological replications were executed. The REST 2009 software^67^ was utilized to calculate the relative gene expression.

### KOBAS (KEGG Orthology-Based Annotation System) and DAVID (Database for Annotation, Visualization and Integrated Discovery)

KOBAS 3.0 was used to predict the target gene-related pathways and their functions in carrot response to salt stress, to identify the significantly enriched GO terms and pathways, and to analyse the metabolic and signalling pathways of these miRNA target genes identify by degradome sequencing. Statistical significance of enrichment was assessed by tool options: statistical method hypergeometric test / Fisher's exact test and FDR Benjamini and Hochberg correction method^68^. Functional term enrichment analysis of miRNA targets was performed using the Functional Annotation Clustering tool in DAVID tool (https://david.ncifcrf.gov/)^69^, applying a medium classification stringency. As an input file a combined list of predicted miRNA targets from psRNATargetScan and degradome were used.

### Ethics approval and consent to participate

The study complies local, Polish and international regulations.

### Supplementary Information

Supplementary Information 1.

Supplementary Information 2.

Supplementary Information 3.

Supplementary Information 4.

Supplementary Information 5.

Supplementary Information 6.

Supplementary Information 7.

Supplementary Information 8.

Supplementary Information 9.

Supplementary Information 10.

Supplementary Information 11.

## Data Availability

The raw datasets used analysed during the current study are available in a SRA database under PRJNA883012 BioProject. Accession numbers are listed in the Supplementary file 10. To obtain data from the SRA database, enter one of the numbers listed in Supplementary File 11 (column A) into the search window. All the other data supporting the results of this article are included within the paper and its supplementary file as figures or tables.
