# Supplementary material for: Comparative analysis of the carrot miRNAome in response to salt stress
Source: Sci Rep. 2023 Dec 6;13:21506. doi: 10.1038/s41598-023-48900-0 (PMC10700493; doi:10.1038/s41598-023-48900-0)
Supplement: Supplementary file 2 — Supplementary Information 2. [file 41598_2023_48900_MOESM2_ESM.pdf]

Comparative analysis of the carrot miRNAome in response to salt stress

Kamil Szymonik, Magdalena Klimek-Chodacka, Aneta Lukasiewicz Alicja Macko-Podgórni, Dariusz Grzebelus, Rafal Baranski

Department of Plant Biology and Biotechnology, Faculty of Biotechnology and Horticulture, University of Agriculture in Krakow, AL. Mickiewicza 21, 31-120, Kraków, Poland

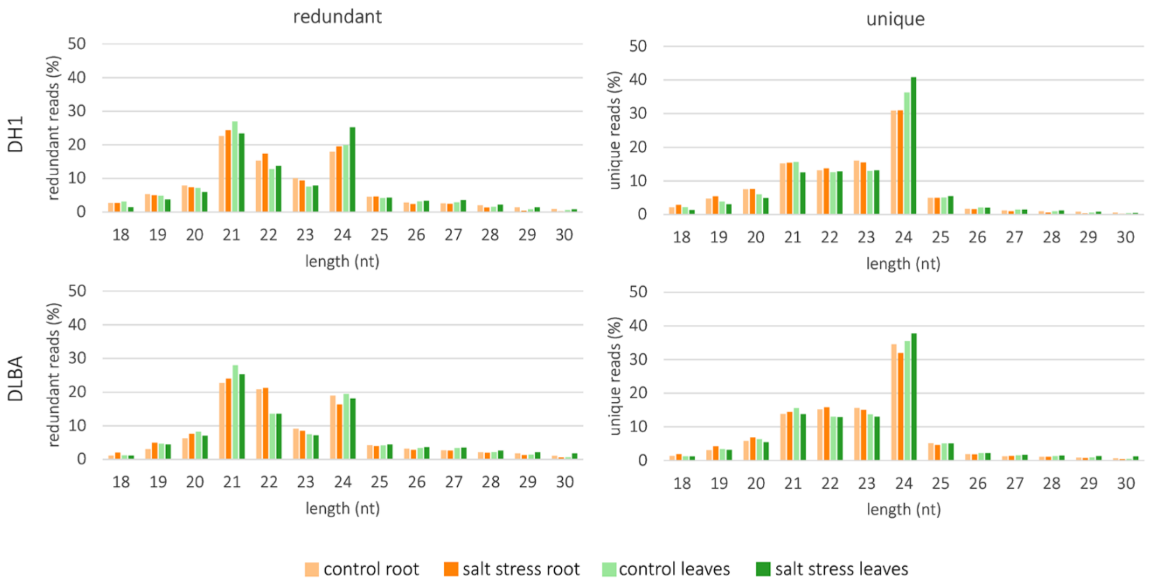

Supplementary file 2 Size distribution of small RNAs in leaves and roots of two carrot varieties (DH1 and DLBA). Redundant (left) and unique (right) sequences identified from the control and salt-treated carrot plants and expressed as percentage of 18 to 30 nt total reads.
