# Supplementary material for: Comparative analysis of the carrot miRNAome in response to salt stress
Source: Sci Rep. 2023 Dec 6;13:21506. doi: 10.1038/s41598-023-48900-0 (PMC10700493; doi:10.1038/s41598-023-48900-0)
Supplement: Supplementary file 4 — Supplementary Information 4. [file 41598_2023_48900_MOESM4_ESM.pdf]

Comparative analysis of the carrot miRNAome in response to salt stress

Kamil Szymonik, Magdalena Klimek-Chodacka, Aneta Lukasiewicz Alicja Macko-Podgórni,

Dariusz Grzebelus, Rafal Baranski

Department of Plant Biology and Biotechnology, Faculty of Biotechnology and Horticulture,

University of Agriculture in Krakow, AL. Mickiewicza 21, 31-120, Kraków, Poland

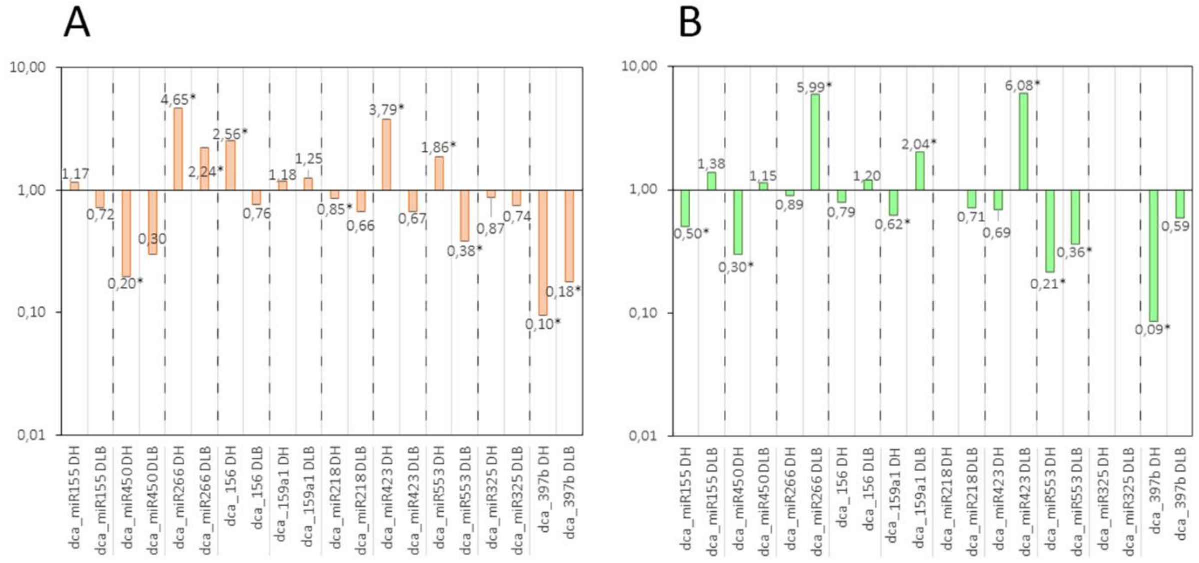

Supplementary file 4 Relative expression level by qPCR of selected conserved and novel dca-miRNA in root (A) and leaves (B). The integrated randomization and bootstrapping methods used in REST 2009 Software test the statistical significance of calculated expression ratios (p-value <0.05). Statistical significant differences are marked by asterisk (*).
